# Bovine α-herpesvirus 1 replication and immediate early transcription unit 1 promoter are jointly stimulated by glucocorticoid receptor and DNA-dependent ATPases

**DOI:** 10.1099/jgv.0.002301

**Published:** 2026-07-15

**Authors:** Fouad S. El-Mayet, Mike St. Clair, Brooke Costello, Jeffery B. Ostler, Clinton Jones

**Affiliations:** 1 Department of Veterinary Pathobiology, College of Veterinary Medicine, Oklahoma State University, Stillwater, OK, USA; 2 Department of Virology, Faculty of Veterinary Medicine, Benha University, Benha, Kaliobyia, Egypt

**Keywords:** bovine herpesvirus 1 (BoHV-1), immediate early transcription unit 1 (IEtu1) promoter, stress-mediated gene expression, SWI/SNF (SWItch/Sucrose-NonFermentable) chromatin remodeling complex, Brahma (BRM) and Brahma-related gene 1 (BRG1) are DNA-dependent ATPases, viral replication and reactivation from latency

## Abstract

Bovine α-herpesvirus 1 (BoHV-1) is a significant viral pathogen in cattle and cofactor of bovine respiratory disease (BRD), a polymicrobial disease. Annually, BRD directly costs the U.S. cattle industry ~$1 billion. Following acute infection, BoHV-1 establishes latency in sensory neurons in trigeminal ganglia, non-neuronal cells in pharyngeal tonsil and certain neurons in the brain. The synthetic corticosteroid dexamethasone (DEX) consistently induces reactivation from latency. The immediate early transcription unit 1 (IEtu1) promoter drives expression of two important viral transcriptional regulatory proteins, infected cell protein 0 (bICP0) and bICP4. An early promoter also drives bICP0 expression. The IEtu1 promoter is activated by the glucocorticoid receptor (GR) and other stress-induced cellular transcription factors because it contains two functional glucocorticoid response elements (GREs). Brahma (BRM) and Brahma-related gene 1 (BRG1) are associated with the SWItch/Sucrose-NonFermentable chromatin remodelling complex. Since BRM and BRG1 interact with GR, we hypothesized that BRG1 and/or BRM stimulate viral gene expression and replication. GR and BRG1 or BRM, cooperatively transactivated an IEtu1 *cis*-regulatory module that contains both GREs. BoHV-1 replication was also significantly reduced by a BRG1 and/or BRM siRNA or a specific BRG1 and BRM inhibitor (BRM014). Chromatin immunoprecipitation studies revealed that BRG1 occupies IEtu1 promoter sequences only when cells express GR and are treated with DEX. In summary, these studies revealed that GR and the BRG1/BRM chromatin-remodelling proteins cooperatively transactivated the IEtu1 promoter and stimulated BoHV-1 productive infection.

## Introduction

 Bovine α-herpesvirus 1 (BoHV-1) is a member of the Herpesviridae family and the Alphaherpesvirinae subfamily [1,2]. BoHV-1 continues to be an important viral pathogen in cattle. During acute infection, BoHV-1 induces conjunctivitis, upper-respiratory tract diseases and suppresses cell-mediated immunity, which leads to secondary infections and virus spread [1,5]. Furthermore, BoHV-1 is a cofactor of bovine respiratory disease (BRD), a polymicrobial disease. The ability of BoHV-1 to suppress immune responses is important for BRD because it can lead to fatal bacterial pneumonia. Finally, BoHV-1 infections can induce abortions in cows [6,8]. Numerous BoHV-1 modified-live vaccines are commercially available and generally they prevent disease [6,8]. However, modified-live vaccines can cause serious disease in young calves and abortions in pregnant cows. Artificial conception is frequently used in cattle and may cause complications because BoHV-1, included modified-live vaccines, can be detected in semen.

**Fig. 1.**
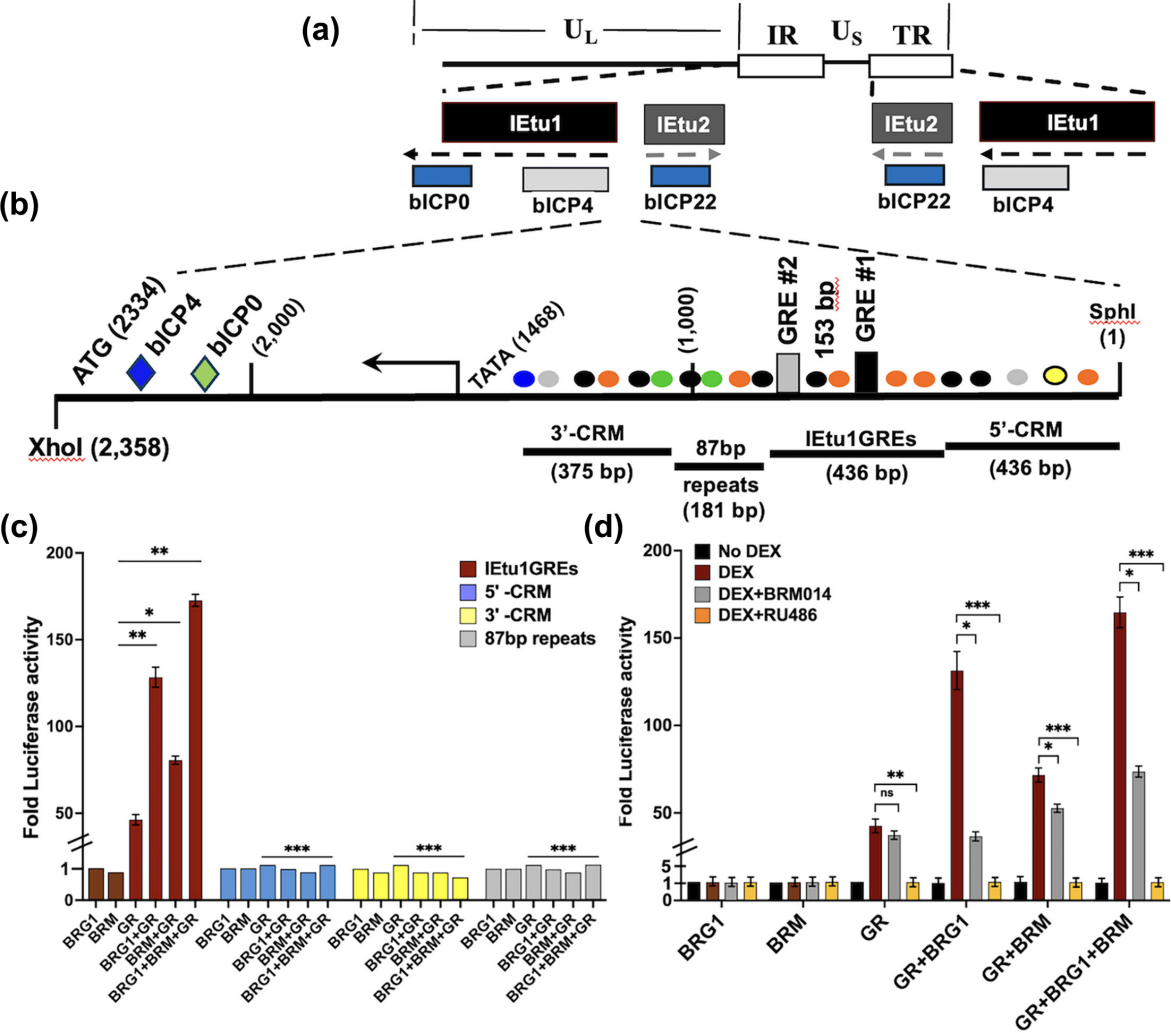
IEtu1 promoter is cooperatively transactivated by glucocorticoid receptor (GR) and BRG1/BRM (Brahma-related gene 1/Brahma gene). (a) Schematic of BoHV-1 genome, internal repeat (IR) and terminal repeat (TR). U_L_ refers to unique long and U_S_ is unique short. A bICP0 E promoter drives expression of the early mRNA bICP0 that is adjacent to the 5′ terminus of bICP0. IEtu2 encodes the bICP22 protein. IEtu1 in the TR encodes only bICP4 because part of bICP0 is contained in U_L_. (b) Schematic of IEtu1 promoter that drives bICP4 and bICP0 immediate early expression. The IEtu1 promoter and IEtu1 *cis*-regulatory module (CRM) constructs were described in a previous publication [27]. Location of GRE#1 (glucocorticoid response element) and GRE#2 in the IEtu1 promoter are denoted by rectangles. Location of key transcription binding sites and start site for transcription of IEtu1 promoter are also presented. The blue diamond points out where bICP4 mRNA is initiated, and the green diamond is where bICP0 transcription is initiated. (c) Neuro-2A cells were transfected with one of the IEtu1 promoter CRM constructs containing the firefly luciferase reporter gene depicted in panel (a) (0.5 µg DNA), the GR-α expression plasmid (0.5 µg DNA), and plasmids that express BRG1 and/or BRM (0.5 µg DNA). All samples contained a plasmid that expresses *Renilla* luciferase (0.05 µg DNA). To maintain the same amount of DNA in each sample, empty vectors were included in certain samples. At 48 h after transfection, cells were harvested and the protein lysate was subjected to a dual-luciferase assay. Asterisks denote a significant difference between the control (IEtu1GREs construct cotransfected only with BRG1) when compared to the denoted IEtu1 promoter CRM construct cotransfected with GR, GR+BRG1, GR+BRM or GR+BRG1+BRM and treated with DEX. The Student’s t-test; *, *P*<0.05, **, *P*<0.01 and *** denotes a significant reduction (*P*<0.001) of the 5′-CRM, 87 bp repeats and 3′-CRM promoter activities relative to the IEtu1GREs construct. (d) Neuro-2A cells were cotransfected with GR-α expression construct, GR+BRG1, GR+BRM and where denoted treated with DEX, RM014 or RU486. At 48 h after transfection, cells were harvested, and the protein lysate was subjected to a dual-luciferase assay as described in the Methods. Asterisks denote a significant difference between the control and samples transfected with the GR, GR+BRG1, GR+BRM or GR+BRG1+BRM and treated with DEX, RM014 or RU486 using the Student’s t-test (ns, non-significant; *, *P*<0.05 and **, *P*<0.01). *** denotes a significant reduction (*P*<0.001) relative to the IEtu1GREs construct cotransfected with BRG1, BRM and DEX treatment. For panel (d), promoter activity in the denoted IEtu1 CRM constructs transfected with BRG1 but not treated with DEX. The results shown in panels (c) and (**d)** are the average of three independent experiments and error bars denote the se. All cultures in panels (c) and (**d)** were incubated with 2% stripped FBS after transfection, and all cultures were treated with DEX 24 h after transfection. The 5′-CRM, 3′-CRM and 87 bp repeats exhibited basal activity (denoted as 1). Since it was difficult to see basal luciferase values of the respective CRM constructs, the Y-value of 1 in panels** (c)** and (**d)** was expanded to visualize basal values.

Robust viral replication occurs during acute infection, which culminates in apoptosis and inflammation [2,9, 10]. Viral gene expression has three distinct phases during productive infection: immediate early (IE), early (E) and late (L). The BoHV-1 immediate early transcription unit 1 (IEtu1) encodes two crucial transcriptional regulators, bICP0 and bICP4 (Fig. 1a and b), that drive productive infection [11,12]. Following acute infection of the oral, nasal or ocular mucosal membranes, latency is established in sensory neurons in trigeminal ganglia (TG) and certain non-neuronal cells within pharyngeal tonsil [13,14]. BoHV-1 genes abundantly expressed in latently infected neurons are the latency related (LR) gene and ORF-E, which is adjacent to the LR gene, reviewed in [15,16]. At least two proteins are encoded by the LR gene (ORF1 and ORF2). Over-expression of the ORF-E protein triggers neuronal differentiation in mouse neuronal cells. ORF2 impairs apoptosis and interacts with several cellular proteins. ORF2 also preferentially binds ssDNA versus dsDNA but does not bind RNA. These ORF2 activities are predicted to play crucial roles during the establishment and maintenance of latency. Finally, small non-coding RNAs encoded by the LR gene impair bICP0 expression [17]. It is unlikely that LR gene products directly enhance reactivation from latency because expression of LR gene products is inhibited by dexamethasone (DEX), a synthetic corticosteroid, reviewed in [18,20]. In contrast to BoHV-1, it is not clear if the herpes simplex virus 1 latency-associated transcript encodes a protein important for latency and/or reactivation. In summary, LR gene products are predicted to coopt cellular signalling pathways that mediate the establishment and maintenance of latency.

DEX mimics stress and consistently triggers BoHV-1 reactivation in TG and pharyngeal tonsil [18,20], which elevates the risk of abortion [7] and BRD [21,22]. The IEtu1 promoter contains two consensus glucocorticoid receptor (GR) response elements (GREs) and is activated by DEX [19,23, 24]. Stress-mediated transactivation of the IEtu1 promoter is predicted to facilitate early events of reactivation from latency [25,26]. Identification of cellular factors that cooperate with GR to enhance IEtu1 promoter activity has provided insight into how viral gene expression is induced during early stages of reactivation from latency [27,28]. GR modulates gene expression by recruiting coregulatory complexes to specific GREs [29], including chromatin remodelling [30,32]. Alternative splicing of the GR mRNA yields GR-α and GR-β. Generally, GR-α but not GR-β transactivates promoters containing GREs and binds DEX or cortisol, reviewed in [33].

Notably, the SWI/SNF (SWItch/Sucrose-NonFermentable) chromatin remodelling complex interacts with GR, which enhances hormone-induced transcription [34]. Brahma (BRM) and Brahma-related gene 1 (BRG1) are DNA-dependent ATPases and subunits of the SWI/SNF family of chromatin-remodelling complexes [35,36]. BRG1 associates with multiple transcriptional coactivators [30,31, 37] and facilitates GR-mediated hormone-induced transcription by various mechanisms [32]. Loss of BRG1/BRM significantly reduces GR-dependent gene expression [38]. BRG1/BRM can also precede GR binding to chromatin by recruiting pioneer factors and remodelling silent chromatin, which enhances GR-mediated transcription [38,41].

Based on these observations, we hypothesized that BRG1 and BRM cooperate with GR to transactivate the IEtu1 promoter, thereby facilitating BoHV-1 gene expression and viral replication. The goals of this study were to test whether GR and BRG1 or BRM cooperatively activate IEtu1 promoter activity and stimulate viral replication.

## Methods

### Cells and virus

Murine neuroblastoma (Neuro-2A; ATCC CCL-131) and African Green Monkey kidney cells (COS-7; ATCC CRL-1651) were grown in minimal essential media (MEM) supplemented with 10% fetal bovine serum (FBS). Media contains penicillin (10 U ml^−1^) and streptomycin (100 µg ml^−1^).

A BoHV-1 mutant containing the β-galactosidase (β-Gal) gene in place of the viral gC gene was obtained from Dr S. Chowdhury [LSU School of Veterinary Medicine (BoHV-1 gCblue virus)], and virus stocks were grown in Madin-Darby Kidney cells. The gCblue virus grows to titres like the wt parental virus and expresses the LacZ gene. Procedures for preparing viral genomic DNA were described previously [42].

### Plasmids

pCMV5 BRGI-Flag was a gift from Joan Massague [43] (Addgene #19143). pBABE hBRM was a gift from Robert Kingston [44] (Addgene #1961). IEtu1 constructs shown in Figs1  2 were inserted into the pGL4.23[luc2/minP] vector (Promega; Madison WI) between the unique *Sac*I and *Hind*III restriction enzyme sites. The pGL4.23[luc2/minP] vector contains a minimal TATA-box promoter element upstream of the luciferase reporter gene and downstream of the multiple cloning region. IEtu1 *cis*-regulatory modules (CRMs) were cloned immediately upstream of the TATA box. All IEtu1 fragments were synthesized by GenScript (Piscataway, NJ).

**Fig. 2.**
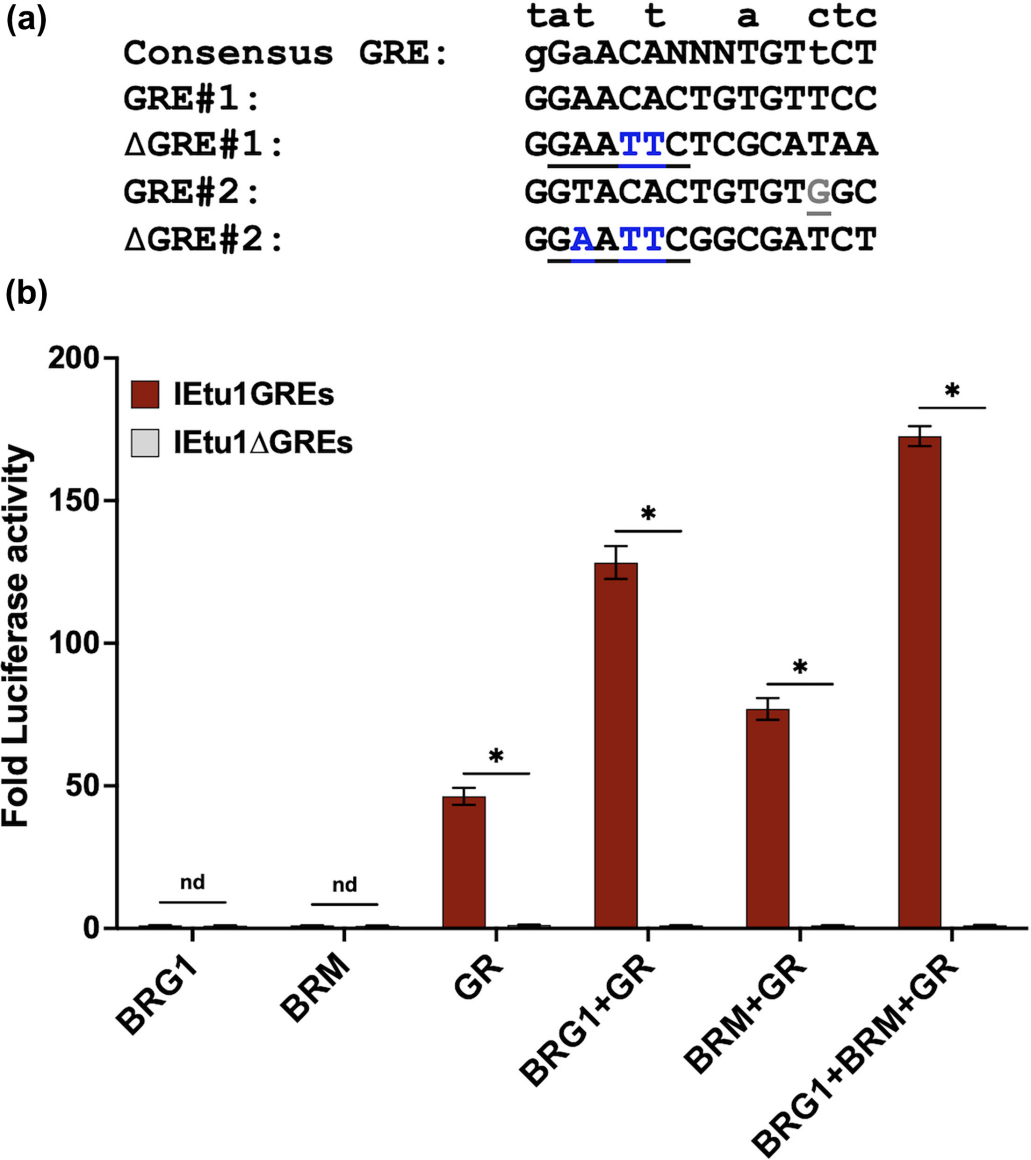
Mutating both GREs in IEtu1 promoter eliminates GR and BRG1/BRM mediated transactivation. (a) The sequence of a consensus GRE [23]. Small letters above a capital letter denote that this nucleotide is less preferred than nucleotides that are capitalized. Two small letters denote that both nucleotides are present in certain consensus sequences but are less preferred relative to capital letters. N is any nucleotide. Nucleotides used to mutate the GREs are an EcoRI site (GAATTC): blue nucleotides denote differences in GRE#1 and GRE#2. The underlined grey nucleotide in GRE#2 is a mismatch of the consensus GRE. (b) Neuro-2A cells were transfected with IEtu1GRE or IEtu1∆GRE constructs containing the firefly luciferase reporter gene (0.5 µg DNA), the GR-α expression plasmid (0.5 µg DNA), BRG1 or BRM (0.5 µg DNA). All samples contained a plasmid that expresses *Renilla* luciferase (0.05 µg DNA), and cultures were treated with 2% ‘stripped’ FBS. Water-soluble DEX (100 nM) was added at 24 h after transfection in certain cultures. Forty-eight hours after transfection, cells were harvested, and protein lysate was subjected to a dual-luciferase assay as described in the Methods. Promoter activity in the denoted IEtu1 CRM constructs inserted into the pGL4.23[luc2/minP] vector was normalized to a value of 1 and fold activation for other samples. The results are the average of three independent experiments, and error bars denote the se. Asterisks denote a significant difference between the control and samples transfected with the GR-α, GR+BRG1 or GR+BRM and treated with DEX using the Student’s t-test (*, *P*<0.05).

A mouse GR-α expression vector was obtained from Dr Joseph Cidlowski, NIEHS, Research Triangle Park, North Carolina. Plasmids were prepared from bacterial cultures by alkaline lysis and two rounds of cesium chloride centrifugation.

### Transfection and dual-luciferase reporter assay

Neuro-2A cells (6×10^5^) were seeded into 60 mm dishes containing MEM (purchased from Corning, 10370021) supplemented with 10% FBS 24 h prior to transfection. Cells were cotransfected with the designated IEtu1 CRM constructs (0.5 µg plasmid DNA) and a plasmid encoding *Renilla* luciferase under the control of a minimal herpesvirus thymidine kinase (TK) promoter (50 ng DNA). To maintain equal plasmid amounts in the transfection mixtures, empty expression vector (pcDNA3.1) was added. Neuro-2A cells were incubated in MEM containing 2% charcoal-stripped FBS obtained from Sigma-Aldrich (F6765) after transfection. Stripped FBS is generated by passing FBS through a column containing ‘activated’ charcoal that removes hormones, certain growth factors and cytokines. Cell cultures were treated with water-soluble DEX (100 nM) (D2915; Sigma-Aldrich), BRM014 (100 nM) (10–5,244; Focus Biomolecules) or RU486 (100 nM) (M8046; Sigma-Aldrich). At 48 h after transfection, cells were harvested and protein extracts were subjected to a dual-luciferase assay using a commercially available kit (E1910; Promega). Luminescence was measured using a GloMax 20/20 luminometer (E5331; Promega).

### Analysis of BRG1/BRM-specific inhibitor and siRNAs on BoHV-1 productive infection

Neuro-2A cells grown in six-well plates were cotransfected with 1.0 µg of the gCblue viral genome and increasing concentrations of a plasmid expressing mouse GR (0.5, 1.0 or 2.0 µg) using Lipofectamine 3000 (L3000150; Invitrogen). Twenty-four hours after transfection, DEX (100 nM) (D2915; Sigma-Aldrich) or BRM014 (100 nM) (10–5244; Focus Biomolecules) were added to transfected cultures to examine the effects on productive infection.

Increasing concentrations of ON-TARGETplus SMARCA4 (BRG1) siRNA (L-010431-00-0005; Dharmacon, GE Healthcare, USA) or ON-TARGETplus SMARCA2 (BRM) siRNA (L-017253-00-0020; Dharmacon, GE Healthcare, USA) (5, 10 and 20 nM) were transfected into Neuro-2A cells and grown in six-well plates using Lipofectamine 3000. Transfected cells were incubated at 37 °C and 5% CO_2_ for 48 h. Cells were collected and lysed with RIPA (Radio-Immunoprecipitation Assay) buffer. Proteins were separated in a 10% SDS-PAGE, and Western blot analysis was performed using anti-BRG1 or anti-BRM antibodies.

Neuro-2A cells were incubated in MEM containing 2% stripped FBS and transfected with scrambled, BRG1 and/or BRM siRNA (20 nM siRNA) using Lipofectamine 3000. A universal scrambled negative-control siRNA (SR30004; Ori-Gene Technologies) was used for certain studies. At 48 h after transfection, cells were cotransfected with BoHV-1 gCblue genomic DNA (1.5 µg) alone or with plasmid expressing mouse GR (2.0 µg). Twenty-four hours after transfection, DEX (100 nM) was added to transfected cultures. Forty-eight hours after transfection, cells were fixed with a solution containing 2% formaldehyde and 0.2% glutaraldehyde in PBS and then stained with a solution containing 1% Bluo-Gal, 5 mM potassium ferricyanide, 5 mM potassium ferrocyanide and 0.5 M MgCl2 in PBS.

The number of β-Gal-positive cells from 10 fields of view was counted, and the mean was calculated. To calculate the fold change of β-Gal cells, the number of blue cells in treated cultures was divided by the number of blue cells in cultures treated with solvent or scrambled siRNA. Results are expressed as fold induction relative to controls because this minimized differences in cell density, Lipofectamine 3000 lot variation and transfection efficiency [45,46].

### Indirect immunofluorescence analysis

Immunofluorescence was performed as previously described in [23,27]. Neuro-2A cells were seeded into two-well chamber slides (177380, Nunc. Inc.) and incubated in MEM supplemented with 10% FBS. Cells were transfected with the GR expression construct or BRG1 expression plasmids for 36 h. Cells were treated with solvent or DEX for 45 min, fixed in 4% paraformaldehyde in PBS pH 7.4 for 10 min at room temperature and permeabilized with 0.25% Triton X-100 in PBS pH 7.4 for 10 min at room temperature. Blocking buffer was performed using 1% BSA in PBST (PBS+0.1% Tween 20) for 30 min to impair non-specific binding of the primary antibody. This step was followed by incubation with anti-GR antibody (3660S; Cell Signaling) and anti-BRG1 antibody (72182S; Cell Signaling) at a concentration of 5 µg ml^−1^ in 1% BSA in PBST overnight at 4 °C. After three washes with PBS, cells were incubated with Alexa Fluor™ 488 goat anti-rabbit IgG (H+L) (A11034; Invitrogen) and Alexa Fluor^®^ 633 goat anti-mouse IgG (H+L) (A21050; Invitrogen) at 1 : 500 dilution for 1 h in the dark. After three washes with PBS, DAPI staining was performed to visualize the nucleus. Slides were covered with coverslips by using VectaShield antifade mounting medium (H-1000; Vector Laboratories). Images were obtained by Cytation 5 Cell Imaging Multimode Reader (Gen5 software, BioTek).

### Co-immunoprecipitation studies and Western blot analysis

Neuro-2A cells were cotransfected with the denoted plasmids that express GR and/or BRG1 (1.5 µg DNA each). Cultures were incubated with MEM containing DEX (100 nM) and 2% stripped FBS for 1 h prior to harvesting transfected cultures. Whole-cell lysate was prepared with RIPA lysis buffer containing 1× Protease Inhibitor cocktail (78429; Thermo Fisher Scientific) and protein concentration quantified. Protein lysate (500 µg) was combined with anti-GR (3660S; Cell Signaling) and/or anti-BRG1 (5 µg) antibodies, and the reactions were incubated overnight at 4 °C on a rotator. Protein A Dynabeads^®^ (Life Technologies) were added and incubated for 2 h at 4 °C with rotation. Immunoprecipitates were collected using a magnet (DynaMag™) (No.12321D, Life Technologies), supernatants were removed and Dynabeads^®^-Ag-Ab complexes were washed three times with 1 ml of washing buffer. Proteins were eluted from Dynabeads^®^ by incubating with 30 µl of elution buffer, and then incubated in a water bath at 42 °C for 30 min. For SDS-PAGE, proteins were mixed with an equal amount of 2× sample loading buffer and boiled for 5 min. Proteins were separated in SDS-10% PAGE gel. After electrophoresis, proteins were transferred onto a PVDF membrane (Immobilon-P; Millipore) and blocked for 1 h in 5% wt/vol. non-fat dry milk with 1× TBS-0.1% Tween 20 (TBS-T). Membranes were incubated with the designated primary antibody at 4 °C with gentle shaking overnight. The primary antibody was diluted 1 : 1,000 in the blocking solution. An antibody directed against GAPDH (Glyceraldehyde 3-phosphate dehydrogenase) (5174S; Cell Signaling) was used as a loading control. After 45 min of washing with TBS-T, blots were incubated with secondary anti-rabbit antibody (7074S; Cell Signaling Technology) or anti-mouse antibody (7076S; Cell Signaling Technology) which was diluted 1 : 2,000 in 5% non-fat milk in TBS-T for 1 h. Blots were washed 45 min with TBS-T and exposed to Amersham ECL reagents, and autoradiography performed.

### Chromatin immunoprecipitation assay

COS-7 cells were grown in MEM supplemented with 10% FBS until 80% confluency. Four hours prior to transfection, cells were washed with PBS and MEM supplemented with 2% charcoal-stripped FBS. Cells were transfected with BoHV-1 genomic DNA, alone or with a GR expression plasmid, using TransIT-X2 (MIR6005; Mirus) transfection reagent. Empty vector plasmid was added where needed to maintain consistent DNA concentrations. At 24 h following transfection, samples were treated with water-soluble DEX. At 48 h later, cells were washed with PBS, formaldehyde fixed and harvested in lysis buffer (50 mM HEPES-KOH, pH 7.5; 140 mM NaCl; 1 mM EDTA; 1% Triton-X; 0.1% sodium deoxycholate; 0.1% SDS). Cell lysate was processed in an ultrasonicator (QSonica) to generate ~500 nt fragments followed by centrifugation to remove cell debris. Chromatin immunoprecipitation (ChIP) was performed as previously described in [20,47, 48]. In brief, 50 µl input samples were removed and set aside. Individual lysate was split into three samples and incubated overnight in RIPA buffer (50 mM Tris/HCl, pH 8.0; 150 mM NaCl; 2 mM EDTA; 1% NP-40; 0.5% sodium deoxycholate; 0.1% SDS) containing anti-GR antibody (3660S; Cell Signaling), anti-BRG1 antibody (72182S; Cell Signaling), or non-specific rabbit IgG (ab171870; Abcam) at 4 °C with rotation. Antibody–protein–DNA complexes were precipitated using Protein A Dynabeads (Invitrogen 1000D), and washed five times: twice with a low-salt buffer (20 mM Tris/HCl, pH 8.0; 150 mM NaCl; 2 mM EDTA; 1% Triton-X; 0.1% SDS), twice with a high-salt buffer (20 mM Tris/HCl, pH 8.0; 500 mM NaCl; 2 mM EDTA; 1% Triton-X; 0.1% SDS), and once with a LiCl buffer (20 mM Tris/HCl, pH 8.0; 250 mM LiCl; 1 mM EDTA; 1% NP-40; 1% sodium deoxycholate). Protein–DNA complexes were eluted from the beads (1% SDS; 100 mM NaHCO3) at 30 °C for 30 min, and crosslinking was reversed at 65 °C overnight, with RNaseA and Proteinase K added to samples. DNA was prepared using phenol/chloroform and amplified by PCR. DNA bands were separated on an agarose gel, visualized by UV with ethidium bromide and quantified using Image Lab software (Bio-Rad).

IEtu1 promoter primers are IEtu1 F (5′-TAGCCGCTCCATTCTCTC-3′) and IEtu1 R (5′-AAAAGTGGGGAAGCAGGG-3′) that yields a 218 bp fragment [49]. The bICP0 E promoter primers used for this study are EP-328 F (5′-GCCCCCCCCCAAAAACAC-3′) and EP-328R (5′-CAAGGCGAAACCCCCCAC-3′) that yield a 130 bp product [50]. All primers were purchased from International DNA Technologies.

## Results

### GR and chromatin remodelling proteins (BRG1/BRM) cooperatively transactivate BoHV-1 IEtu1 promoter activity

The IEtu1 promoter drives expression of two IE mRNAs that are generated from an alternatively spliced mRNA, which subsequently encodes bICP0 and bICP4 (see Fig. 1A and B for schematic of IEtu1 and IEtu2) [11,12]. Interestingly, a separate bICP0 E promoter sustains bICP0 throughout productive infection. The bICP0 and bICP4 proteins are crucial for viral replication. Notably, the transcription factor binding sites in the IEtu1 promoter, including two GREs, are conserved in respiratory and genital viral isolates (Fig. 1B) [51].

To test whether BRG1 and BRM regulate IEtu1 promoter activity, dual-luciferase assays were performed using four CRMs that span the IEtu1 promoter (Fig. 1B). The CRM fragments were cloned upstream of a firefly luciferase reporter plasmid that contains a minimal TATA box promoter (pGL4.24[luc2P/minP]) [26]. The IEtu1 CRM constructs were cotransfected into mouse neuroblastoma (Neuro-2A) cells with plasmids that express GR-α, BRG1 and/or BRM, and a *Renilla* luciferase gene driven by a minimal TK promoter. Neuro-2A cells were used for these studies because they resemble dopamine-like neurons after differentiation [52] and ~50% of Neuro-2A cells are transfected [20].

The rationale for examining the effects of GR-α and DEX or BRG1 or BRM is that these transcription factors (BRG1 and BRM) mediate interactions with chromatin and pioneer factors [38]. Furthermore, GR and certain stress-induced cellular factors efficiently transactivate IEtu1 promoter activity [20,27, 28]. After transfection, Neuro-2A cells were incubated in MEM that contains 2% stripped FBS. BoHV-1 does not efficiently grow in Neuro-2A cells [53], in part because the endogenous GR expressed in Neuro-2A cells is smaller than GR-α and does not efficiently transactivate GR-responsive promoters [22,53]. Thus, for all studies, a mouse GR-α expression plasmid was used and hereafter is denoted as the GR construct.

For the studies presented in Fig. 1c, cells were treated with DEX after transfection, as described in the Methods. When Neuro-2A cells were transfected with the IEtu1 GREs construct and plasmids that express BRG1 or BRM, promoter activity was negligible (Fig. 1c). GR +DEX transactivated the IEtu1GREs CRM by 46-fold in Neuro-2A cells, consistent with our previously published studies [27]. Cotransfection of GR with BRG1 or BRM cooperatively transactivated the IEtu1GREs CRM construct in Neuro-2A cells, resulting in 128-fold or 80-fold induction versus basal activity. When GR was cotransfected with BRG1 and BRM, IEtu1GREs CRM transcriptional activity was increased ~172-fold when cultures were treated with DEX. In contrast, the other three IEtu1 CRM constructs (3′-CRM, 5′-CRM or the 87 bp repeat) were not transactivated by BRG1, BRM and GR, regardless of whether cultures were treated with DEX. These three constructs (3′-CRM, 5′-CRM and the 87 bp repeat) lack a consensus GRE, which is important for BRG1 and/or BRM-mediated transcription [32]. Consequently, it was not surprising that GR, BRG1 and BRM did not increase promoter activity of the three CRM constructs lacking a GRE, and their promoter activity was significantly lower when compared to the IEtu1 GREs results.

**Fig. 3.**
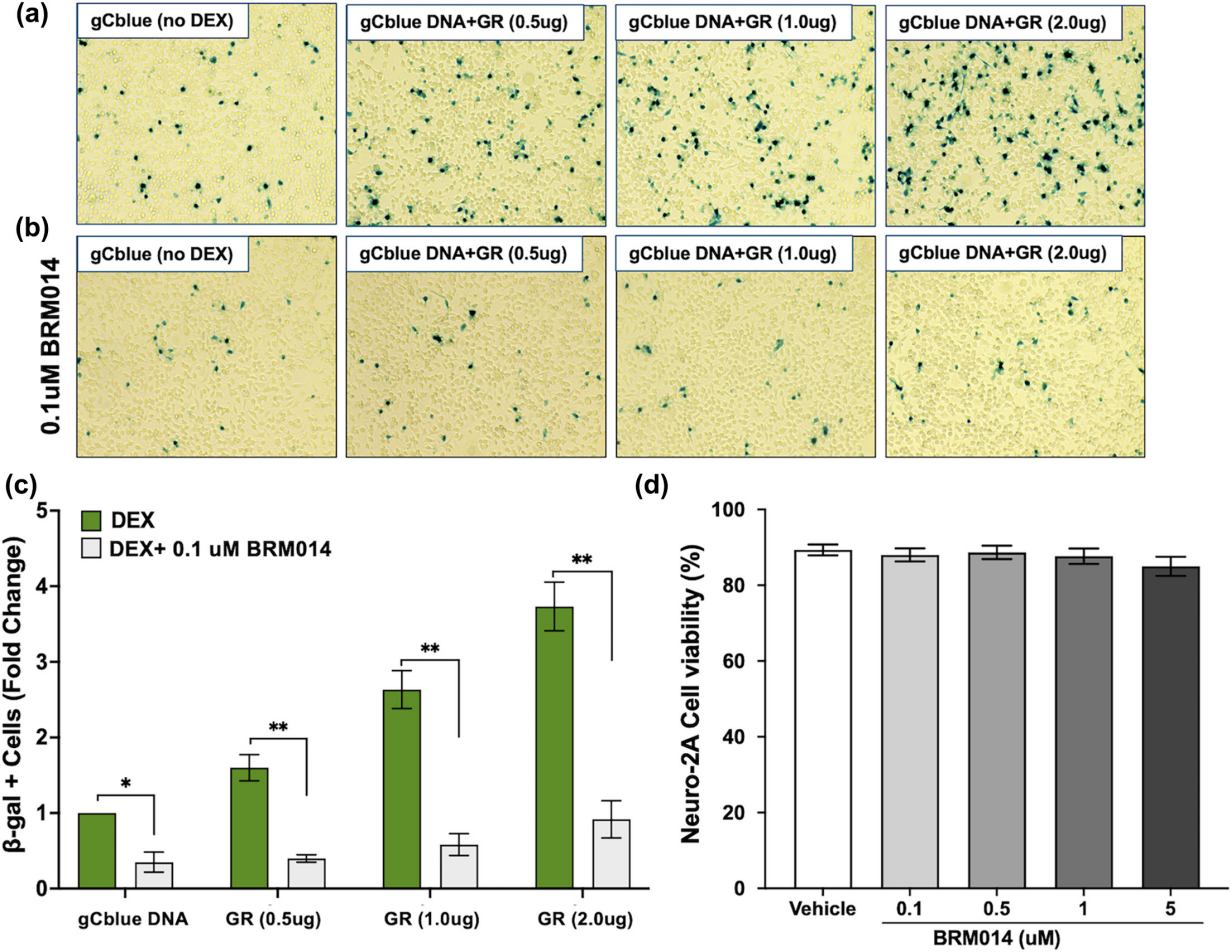
BRM014 disrupted the ability of BRG1/BRM to significantly reduce the effect that GR-α has on BoHV-1 replication. Neuro-2A cells were transfected with BoHV-1 gCblue genomic DNA (1.0 µg) plus increasing amounts of plasmid expressing the mouse GR-α (0.5, 1.0 or 2.0 µg). To equalize total DNA, empty vector was added where necessary. Twenty-four hours post-transfection, cells were incubated with 2% stripped FBS and treated, as indicated, with an empty plasmid and DEX (a) or GR-α, DEX and/or BRM014 (0.1 uM) (b). For controls, DEX was not treated with certain cultures (no DEX). β-Gal-positive cells were quantified 48 h post-transfection. For normalization, the value for the control (gCblue DNA alone) was set to 1. Data represent the mean of three independent experiments. (c) Quantification of fold changes in β-Gal-positive cells. Statistical significance between DEX versus DEX+BRM014 treatments was determined by Student’s t-test (*, *P*<0.05 or **, *P*<0.01). (d) Neuro-2A cells were grown in MEM containing 5% FBS with increasing concentrations of BRM014 as indicated for 24 h. Cytotoxicity was determined using the trypan blue exclusion assay with the Bio-Rad TC20 automated cell counter. Data are shown as sem for triplicate wells of duplicate experiments.

BRM014 is a specific inhibitor of BRG1 and BRM chromatin remodelers [54]. Inhibiting BRG1/BRM ATPase activity by BRM014 reduces chromatin accessibility and BRG1/BRM-dependent gene expression [55]. As expected, treatment of transfected cells with BRM014 significantly reduced transactivation of the IEtu1GREs construct mediated by GR+BRG1, GR+BRM or GR+BRG1+BRM to levels observed with GR+DEX alone (Fig. 1d). BRM014 cytotoxicity in Neuro-2A cells was not significant at concentrations up to 5 µM (data not shown and see Fig. 3d). Treating cultures with RU486, a GR and progesterone receptor antagonist [56], reduced transactivation to basal activity of the IEtu1GREs CRM. In contrast to the IEtu1 GREs construct, GR, BRG1 and BRM did not cooperatively transactivate the bICP0 E promoter (El-Mayet, unpublished studies). Collectively, these results revealed that only the IEtu1GREs CRM construct was cooperatively transactivated by BRG1, BRM and GR in DEX-treated Neuro-2A cells.

### Mutating GREs in the IEtu1GREs CRM construct inhibited the effects of GR, BRG1 and BRM

Previous studies demonstrated that mutating the two consensus GREs in the IEtu1 promoter reduced GR-mediated promoter activity to basal promoter levels [20,23, 24]. Sequences within the IEtu1 GREs sequences contain 2 consensus GREs and 4½ GREs. Mutating GRE#1 or GRE#2 significantly reduces promoter activity in transient transfection studies relative to the construct containing both GREs. Furthermore, mutating GRE#1 generally had increased reduction when compared to just mutating GRE#2 in Neuro-2A cells. Although mutating the 4½ GREs significantly reduced promoter, mutating GRE#1 and GRE#2 essentially reduced promoter activity to baseline. Hence, the rationale for these studies was to determine if the GR expression construct, BRG1 and/or BRM efficiently stimulated promoter activity of the wt IEtu1GREs construct. To determine if the GREs were essential for transactivation by GR, BRG1 and BRM, promoter activation of the wt IEtu1GREs was compared to a construct containing both mutant GREs that is referred to as IEtu1∆GRE (Fig. 2a). Cotransfection of Neuro-2A cells with BRG1, BRM, GR and DEX treatment failed to cooperatively transactivate the IEtu1∆GREs construct (Fig. 2b). These studies demonstrated that the consensus GREs in the IEtu1GREs CRM construct were essential for cooperative transactivation by GR, BRG1, BRM and DEX treatment.

### BRG1/BRM enhance BoHV-1 replication in cultured neuronal cells

BRG1 interacts with multiple transcriptional coactivators and facilitates GR-mediated hormone-induced transcription [38]. To test whether BRG1/BRM and GR regulate BoHV-1 productive infection, Neuro-2A cells were cotransfected with gCblue genomic DNA. Transfecting viral DNA instead of infecting cells was performed because BoHV-1 infectious virus particles contain VP16, bICP4 and bICP27 [57] that can activate viral replication [19,50, 57] and reduce the effects of DEX. A gCblue BoHV-1 recombinant virus has a Lac Z gene downstream of the gC promoter and this virus grows like wt BoHV-1 in bovine cells. The number of β-Gal-positive cells correlates with plaque formation [20,58]. Increasing GR concentrations significantly increased β-Gal+Neuro-2A cells from 1.5- to 3.6-fold if cells were treated with DEX (Fig. 3a). BRM014 treatment significantly reduced the number of β-Gal-positive cells, consistent with BoHV-1 replication (Fig. 3b and c). BRM014 cytotoxicity in Neuro-2A cells was not significant at concentrations up to 5 µM (Fig. 3d).

**Fig. 4.**
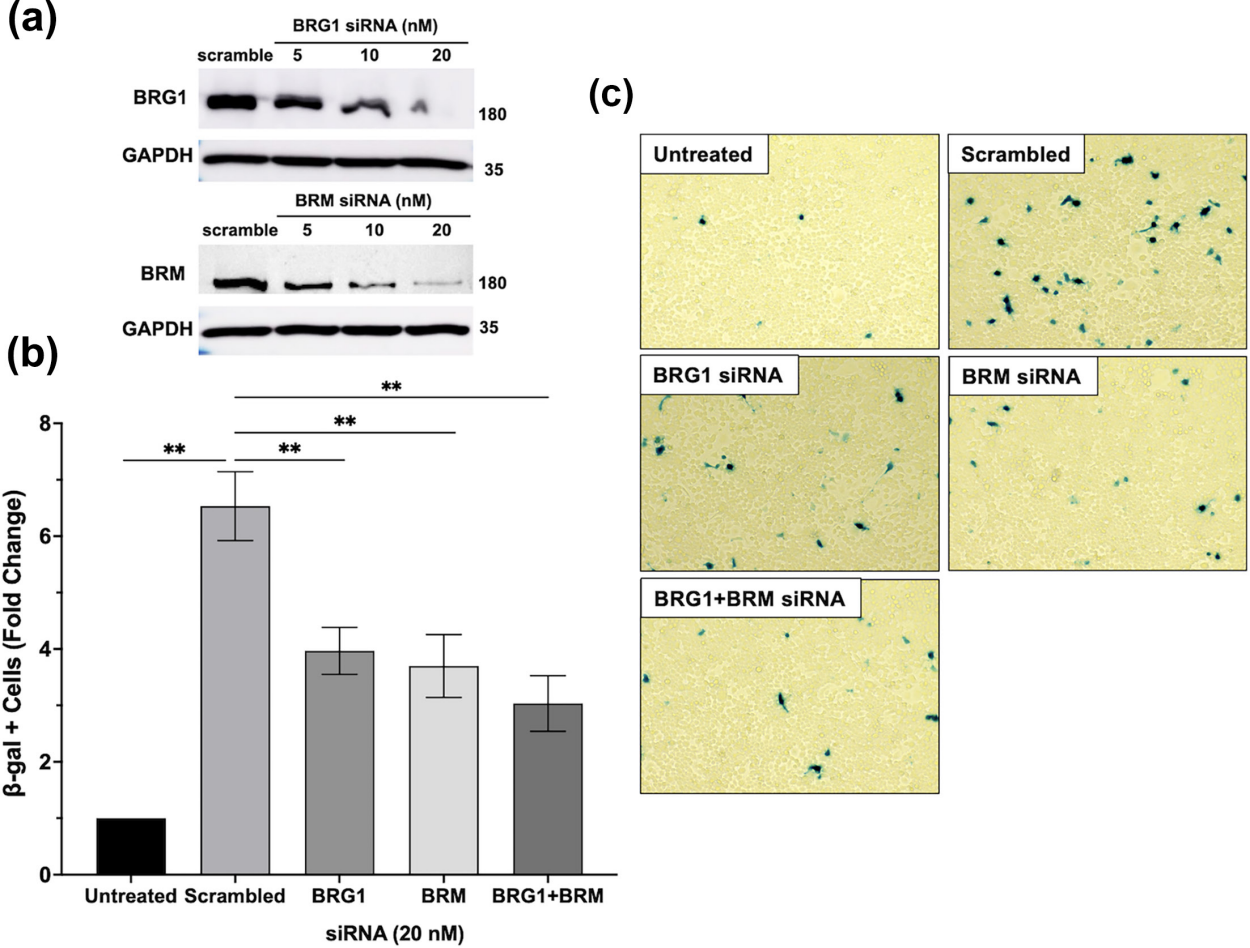
Silencing BRG1 and BRM impairs BoHV-1 productive infection. (a) All samples were transfected with gC blue genomic DNA after silencing with denoted siRNA. Increasing concentrations of BRG1 or BRM siRNA (5, 10 or 20 nM) were transfected into Neuro-2A cells using Lipofectamine 3000 as described in the Methods. Transfected cells were incubated at 37 °C and 5% CO_2_ for 48 h. Cells were collected and lysed with RIPA buffer. Proteins were separated in a 10% SDS-PAGE, and Western blot was performed using a BRG1 or BRM antibody. GAPDH protein levels were examined as a loading control. In four independent studies, the BRG1 or BRM siRNA had no effect on GAPDH levels. (b) Neuro-2A cells were grown in MEM containing 2% stripped FBS and transfected with scrambled siRNA as a negative control, BRG1 and/or BRM siRNA (20 nM siRNA for each) using Lipofectamine 3000 according to the manufacturer instructions. At 48 h after transfection, cells were cotransfected with BoHV-1 gCblue genomic DNA (1.5 µg) and with a plasmid expressing mouse GR-α (2.0 µg). Untreated cells were transfected with gC blue viral genomic DNA but no siRNA or GR-α. At 24 h post-transfection, cells were incubated with 2% charcoal-stripped FBS and treated, as indicated, with DEX (100 nM). β-Gal-positive cells were quantified 48 h post-transfection after staining with β-Gal stain. The value for the control (gC blue DNA cotransfected with empty vector and then treated with PBS after transfection) was set at 1. The results from GR-transfected samples were compared to those from the control and the number of β-Gal+Neuro-2A cells from four independent quadrants/multi-plate were counted. The results in panel (b) are the average of three independent experiments. An asterisk denotes a significant difference between samples transfected with GR+DEX and samples silenced with BRG1 and/or BRM siRNA using the Student’s t-test (**, *P*<0.01). (c) Representative photographs of cultures following transfection with gC blue DNA and GR-α after β-Gal staining was performed.

To confirm the results of BRM014, additional studies tested whether silencing BRG1, BRM or both BRG1/BRM influenced GR-α-induced BoHV-1 replication in transfected Neuro-2A cells (Fig. 4). As expected, BRG1 and BRM protein levels were reduced in cells transfected with the specific siRNA versus a scrambled siRNA (Fig. 4a). However, BRG1 or BRM siRNAs did not reduce GAPDH protein levels. Silencing BRG1 or BRM, and when both siRNAs were used, was a significant reduction in the number of β-Gal-positive cells observed when compared to Neuro-2A cells transfected with a scrambled siRNA (Fig. 4b and c). Collectively, the studies in Figs3  4 revealed BoHV-1 productive infection was cooperatively stimulated by GR and BRG1 or BRM in Neuro-2A cells. Finally, BRM014 treatment or a specific BRG and/or/BRM siRNA significantly reduced BoHV-1 replication.

### BRG1 and GR localize to the nucleus and interact if cells are treated with DEX

**Fig. 5.**
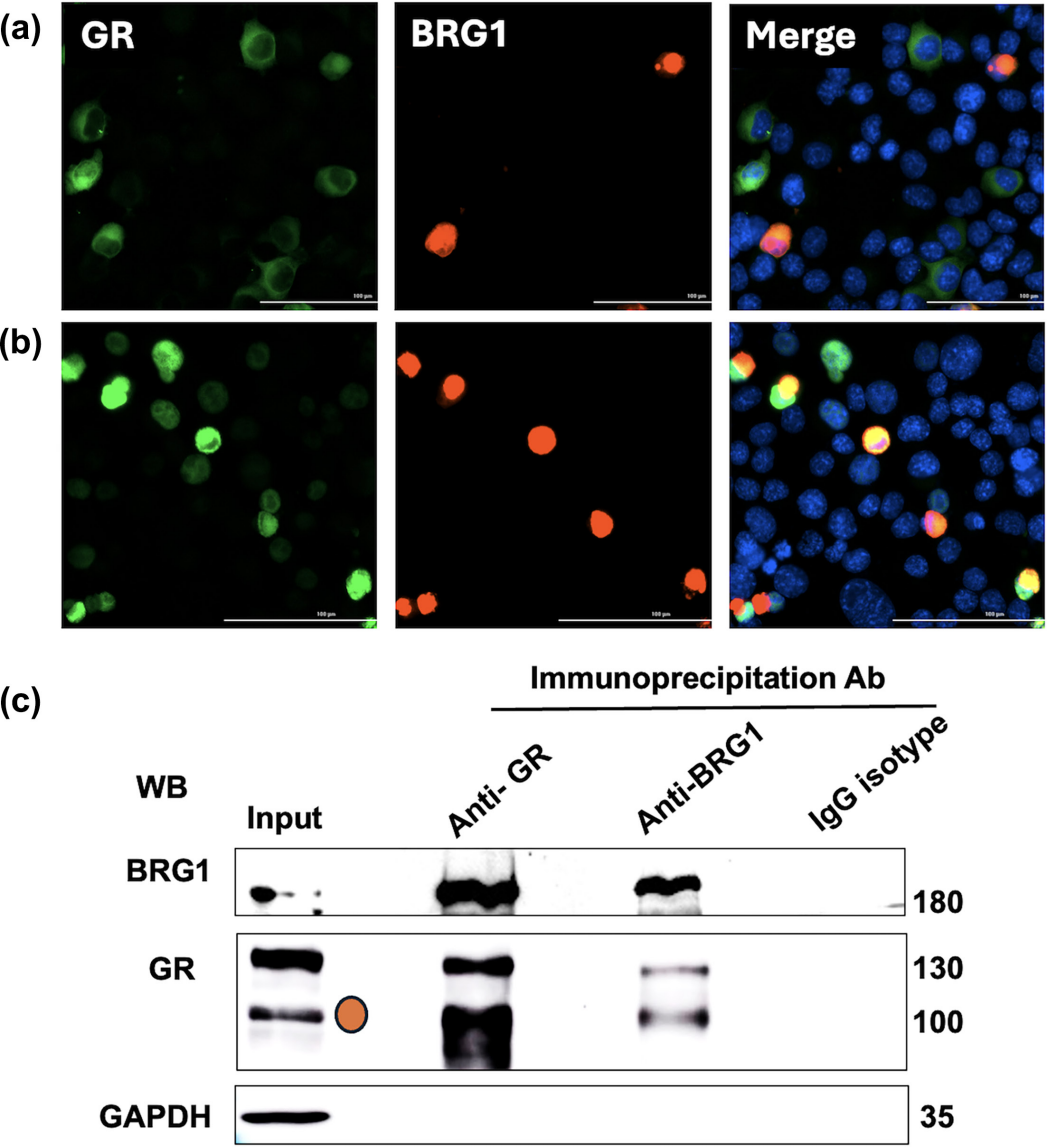
GR and BRG1 are present in the nucleus and physically interact after DEX treatment. Neuro-2A cells were seeded on multi-well cell culture slides and cotransfected with plasmids expressing GR-α and BRG1 in MEM supplemented with 2% stripped FBS for 48 h. Cells were treated with solvent (No DEX) (a) or 100 nM DEX (b) for 1 h followed by indirect immunofluorescence analysis to detect GR-α (green signal) and BRG1 (red signal). Nuclei were visualized using DAPI (blue staining). Images were examined using the BioTek Cytation 5 Cell Imaging Reader, and each panel is 40× magnification and are representative of three independent experiments. Results in panel (c) are the average of counting BRG1-positive cells from three independent experiments. An asterisk denotes a significant difference between control samples (no DEX) and samples treated with DEX using the Student’s t-test (*, *P*<0.01). Neuro-2A cells were grown to 70% confluence on 100 mm dishes. Cells were transfected with a plasmid expressing GR-α and BRG1. Cultures were treated with DEX (100 nM) in MEM containing 2% stripped FBS for 1 h prior to harvesting whole-cell lysate. Co-immunoprecipitation (co-IP) studies were performed using GR- or BRG1-specific antibodies as denoted, or an isotype-specific control antibody described in the Methods. Following immunoprecipitation (IP) with the designated antibody, BRG1 or GR-α was detected in the immunoprecipitate. Input whole-cell lysate (30 µg protein) was used as a positive control. Endogenous GR expressed in Neuro-2A cells is denoted by a red closed circle versus GR-α expressed from the GR expression plasmid. A Western blot was used to detect GAPDH.

To examine GR-α and BRG1 subcellular localization, Neuro-2A cells were cotransfected with plasmids expressing these proteins and co-immunostaining performed. For these studies, cultures were incubated with media containing ‘stripped’ FBS. GR-α was localized in the cytoplasm of control cells incubated with MEM lacking DEX, which is consistent with previous studies [23] (Fig. 5a). Following DEX treatment, GR was localized in the nucleus (Fig. 5b). Although BRG1 was detected in the nucleus regardless of DEX treatment, addition of DEX treatment increased the numbers of cells expressing BRG1 (Fig. 5c). GR-α is known to interact with BRG1 [29,31], suggesting that these interactions stabilize BRG1 levels.

Since SWI/SNF complexes containing BRG1/BRM interact with GR in tissues and cell lines [39,59, 60], studies were performed to test if BRG1 physically interacts with GR. Neuro-2A cells were transfected with plasmids that express GR-α or BRG1, cultures were treated with DEX and co-immunoprecipitation (co-IP) studies were performed. A GR-α expression plasmid was transfected into Neuro-2A cells because GR proteins expressed in Neuro-2A cells are smaller proteins (Fig. 5c, red circle) [28].

Following immunoprecipitation (IP) with the anti-GR antibody, BRG1 was consistently detected in the immunoprecipitate (Fig. 5c). When IP was performed using the anti-BRG1 antibody, GR-α and the endogenous GR were detected in immunoprecipitates (Fig. 5c). As expected, IP with an isotype control antibody did not result in IP of BRG1 or GR. GAPDH was readily detected in whole-cell lysate but was absent from the immunoprecipitants (Fig. 5c), confirming IP procedures removed cell lysate. These findings confirmed GR-α or the smaller GR isoform and BRG1 colocalized in Neuro-2A cells and were associated with each other following DEX treatment.

### BRG1 and GR occupy IEtu1 promoter sequences when BoHV-1 DNA is transfected into COS-7 cells, which do not express GR

GR-α directly interacts with components of the basal transcription machinery, facilitates recruitment of histone-modifying enzymes and chromatin-remodelling complexes, including the SWI/SNF complex [61,62]. As a comparison to Neuro-2A cells, COS-7 cells were transfected with the BoHV-1 genome alone or with the GR-α expression plasmid, with or without DEX treatment. Transfection was chosen to avoid the complication of BoHV-1 virion-associated virus proteins (ICP0, ICP4 and VP16) that are potent transcriptional activators [20,51, 57, 58]. Previous studies demonstrated endogenously expressed GR in Neuro-2A cells bind the virus genome [26,47, 50]. COS-7 and parental CV-1 cells (African Green Monkey Kidney Cells) do not express GR [63] and were chosen to test if BRG1 occupies IEtu1 and bICP0 E promoters in the absence of GR. Although COS-7 cells are non-permissive for BoHV-1 replication, BoHV-1 replicates efficiently when these cells are transfected with the GR-α construct and treated with DEX [48].

**Fig. 6.**
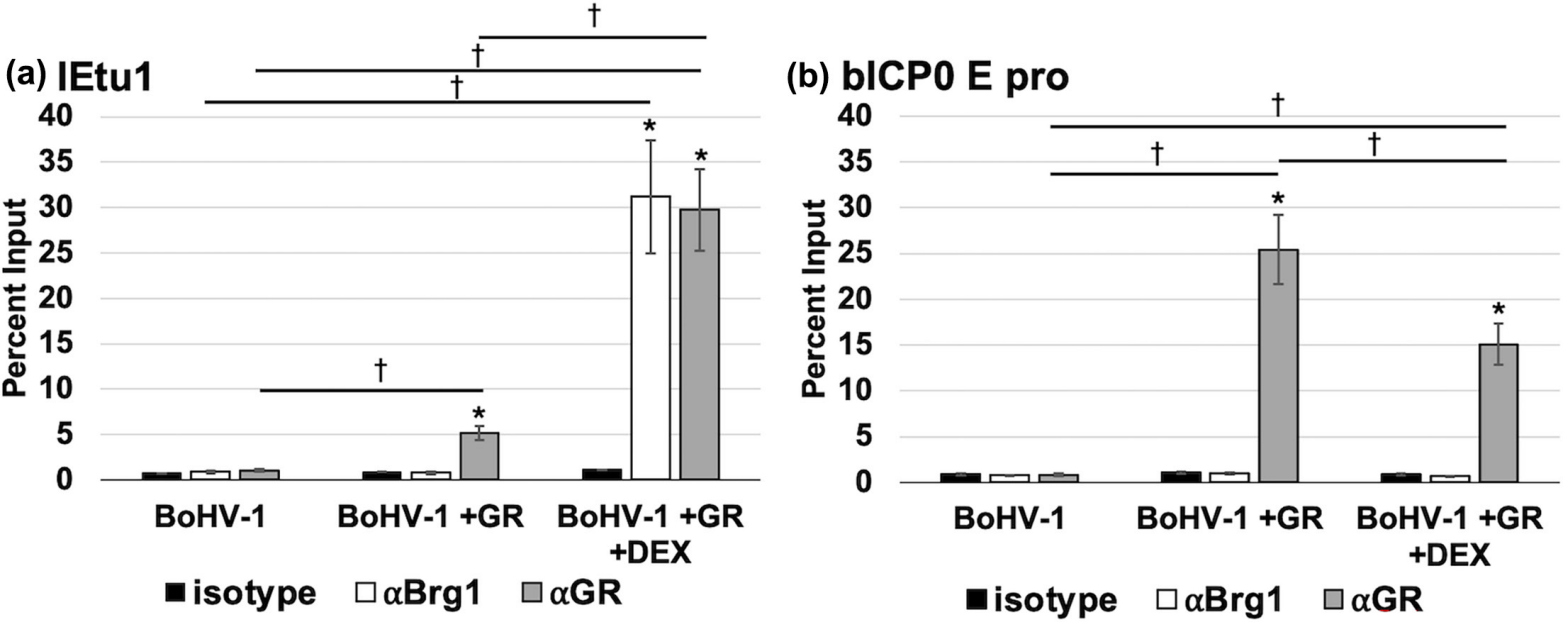
BRG1 and GR occupy IEtu1 promoter sequences in the BoHV-1 genome following transfection of COS-7 cells. COS-7 cells were transfected with the BoHV-1 genomic DNA alone or in combination with the GR-α construct and grown in MEM containing 2% charcoal-stripped FBS. Designated samples were treated with DEX at 24 h post-transfection. At 48 h post-transfection, cells were harvested for ChIP studies as described in the Methods, and immunoprecipitated with non-specific rabbit IgG (isotype control antibody), anti-BRG1 antibody (⍺Brg1) or anti-GR antibody (⍺GR). Precipitated DNA was purified and amplified by PCR using either IEtu1 (a) or bICP0 E promoter (b) primers, as described in the Methods. A statistically significant difference between an antibody-treated sample and isotype is indicated by an asterisk (*). A statistically significant difference between two antibody-treated samples is denoted with a cross (†) above a line connecting the indicated samples. All statistics were performed using a Student’s t-test, with a *P*-value of *P*<0.01 indicating statistical significance. Data are presented as a percentage of the indicated sample relative to the input sample. Error bars are the average of three independent experiments denote the sd.

ChIP studies tested whether GR-α and BRG1 occupied IEtu1 promoter sequences when GR is expressed and DEX treated in COS-7 cells (Fig. 6a). GR-α only occupied the IEtu1 promoter when the viral genome was cotransfected with the GR-α expression construct (Fig. 6a). Furthermore, endogenous BRG1 did not occupy IEtu1 promoter sequences when COS-7 cells were transfected with the GR-α construct. When GR-α was expressed and DEX added to COS-7 cells significantly higher GR levels occupied IEtu1 promoter sequences. Notably, BRG1 occupancy of IEtu1 promoter sequences only occurred when COS-7 cells were transfected with the GR construct and cultures were treated with DEX.

In contrast to the IEtu1 promoter, BRG1 did not occupy bICP0 early promoter sequences when COS-7 cells were cotransfected with the BoHV-1 genome and GR-α expression construct, even if cultures were treated with DEX (Fig. 6b). As expected, GR-α occupied bICP0 early promoter sequences when the GR expression construct was expressed [64]. DEX reduced GR-α bound to the bICP0 E promoter because this promoter is transactivated more efficiently by GR-α without DEX treatment because the promoter is activated via an unliganded mechanism [26]. In summary, these results demonstrated GR-α and DEX were required for BRG1 occupancy of IEtu1 promoter sequences. BRG1 also does not occupy bICP0 early promoter sequences when COS-7 cells were transfected with the GR-α expression plasmid and DEX added.

## Discussion

Stress consistently induces BoHV-1 reactivation from latency in calves [48,65,67]. This is one of the few neurotropic Alphaherpesvirinae subfamily members where the natural host can be used to understand reactivation from latency. Furthermore, the early stages of stress-induced BoHV-1 reactivation from latency can be readily performed in the natural host. Immunohistochemistry studies demonstrated that bICP0, bICP4 and VP16 are readily detected in TG neurons within 30–180 min after DEX treatment of latently infected calves [19,68, 69]. Conversely, two viral glycoproteins (gC and gD) are not readily detected in TG neurons until 6 h after DEX treatment. Since bICP0 and bICP4 are detected in TG neurons during early stages of reactivation from latency, it is reasonable to predict that activation of the IEtu1 promoter is crucial for DEX-induced reactivation from latency. Furthermore, recent studies revealed GR is bound to the IEtu1 and bICP0 E promoter within 3 h after an injection of DEX into the jugular vein to launch reactivation from latency; conversely, GR is not bound to these two viral promoters in TG of latently infected calves [49].

**Fig. 7.**
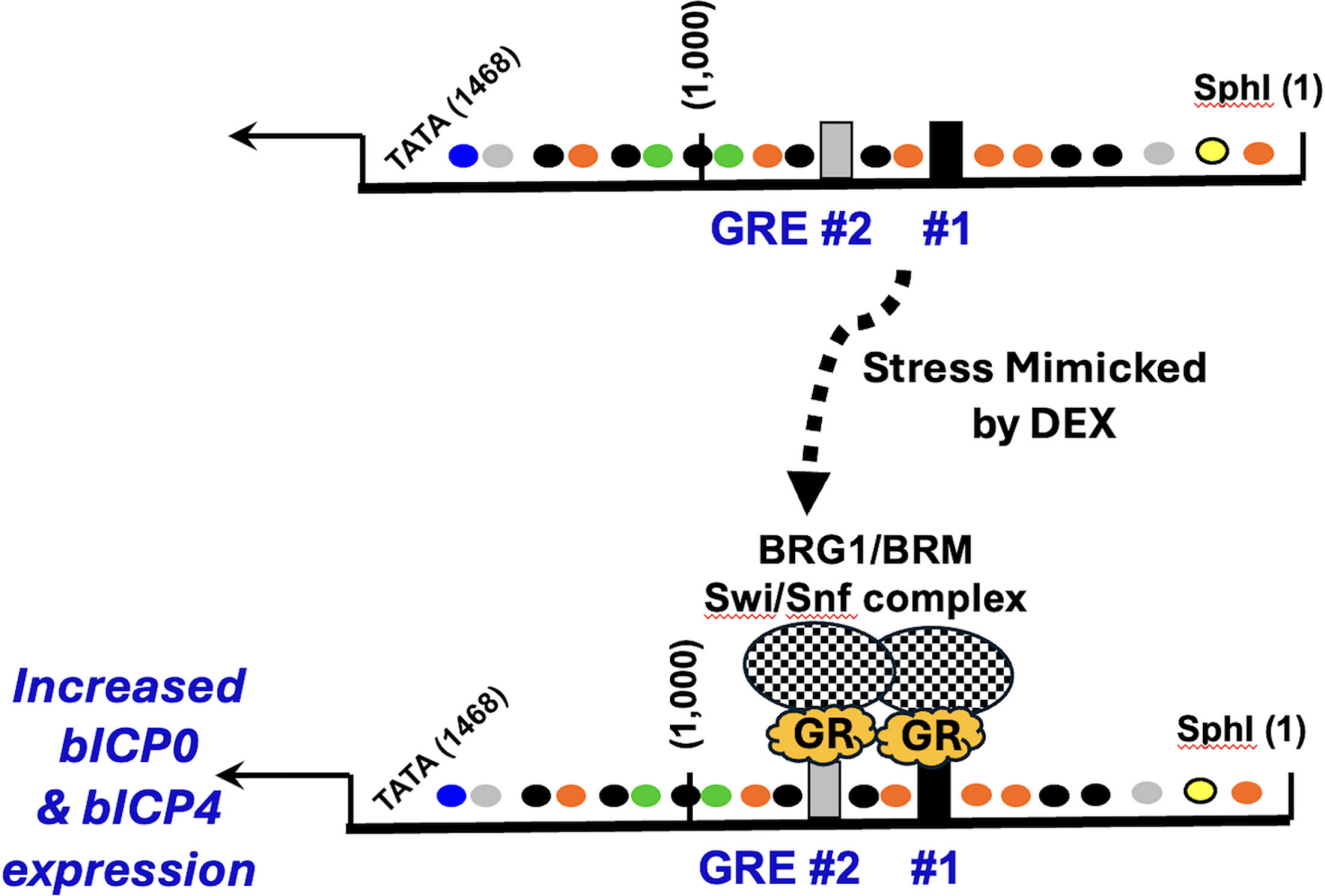
Schematic of stress-mediated GR activation pathways leading to chromatin remodelling and transcription. A GR–hormone complex directly interacts with DNA following a stressful stimuli or treatment with DEX. The GR–hormone complex also recruits a BRG1/BRM and SWI/SNF complex. Additional transcriptional cofactors are likely to be recruited to the GREs in the IEtu1 promoter. Consequently, chromatin remodelling and induction of transcription occurs.

Enhanced stress, as mediated by DEX treatment, leads to GR-α interacting with GRE#1 and #2 (see Fig. 7 for a simple schematic). Consequently, an SWI/SNF complex that contains BRG1 or BRM interacts with GR bound to GRE#1 and GRE#2, which culminates in IEtu1 chromatin remodelling and then induction of transcription (Fig. 7). GR recruitment of BRG1 and/or BRM SWI/SNF complex to IEtu1 promoter occurs after DEX treatment. This is supported by ChIP studies that revealed binding of Brg1 to the IEtu1 promoter in COS-7 cells only when cultures were transfected with GR and treated with DEX. Conversely, a BRG1 complex can occupy sequences of certain promoters adjacent to a GRE and recruit pioneer factors in the absence of GR bound to a GRE, which can also lead to transcriptional activation [29,31, 70]. The BoHV-1 genome contains ~100 consensus GREs [23] suggesting GR, BRG1 and/or BRM activates additional viral promoters during replication or reactivation from latency.

GR contains numerous serine and threonine residues that regulate various GR functions, reviewed in [71]. Although studies have not tested whether phosphorylated GR regulates interactions with BRG1 or BRM, published reports concluded that phosphorylation of GR at serine 203 correlates with localization to the cytoplasm [72]. Mutating serine 211 to alanine induces conformational changes in the GR activation function region 1 [73] suggesting this mutation impairs interactions and cooperative transactivation with BRG1 and/or BRM. Future studies are needed to test whether GR phosphorylation is important for interactions with BRG1/BRM and the IEtu1 promoter.

BRG1 and/or BRM do not mediate GR transactivation of the bICP0 E promoter, presumably because BRG1 does not occupy bICP0 E promoter sequences during infection of COS-7 cells regardless of GR and/or DEX treatment (Fig. 6b). The bICP0 E promoter is cooperatively transactivated by GR and the pioneer factor KLF4, a pioneer transcription factor, via a ligand-independent mechanism [74]. The ½ GREs in the bICP0 E promoter do not stimulate GR- and KLF4-mediated transactivation, suggesting KLF4 and other transcriptional coactivators trigger bICP0 E promoter activity [50,64]. We also suggest that certain external stressors may initially trigger the bICP0 E promoter, whereas increased corticosterone preferentially induces IEtu1 promoter activity.

Although TG neurons are primary sites for latency, BoHV-1 also establishes a latent infection in certain pharyngeal tonsil cells and DEX initiates reactivation from latency [25]. In contrast to TG neurons, LR gene expression is not detected in pharyngeal tonsil. Interestingly, bICP0 and VP16 are expressed in TG neurons prior to bICP4 during DEX-induced reactivation [69]. In contrast, 30 min after DEX treatment of latently infected calves, bICP4 RNA is detected in pharyngeal tonsil, but bICP0 RNA is not detected, suggesting that splicing of the IEtu1 transcript in pharyngeal tonsil only produces bICP4 expression [75]. By 90 min after DEX treatment, low levels of bICP0 RNA are detected and 3 h after DEX treatment all viral transcripts are expressed. Thus, DEX treatment induces expression of all viral genes in certain pharyngeal tonsil cells faster than TG neurons, and VP16, bICP0 and bICP4 expression is different during reactivation from latency. Furthermore, bICP0 expression appears to be more important in certain cells, whereas bICP4 is more important in other cell types. Future studies will compare BRG1 interactions with the IEtu1 promoter and splicing of the IEtu1 mRNA during reactivation from latency in TG versus pharyngeal tonsil.

## Abbreviations

BoHV-1: bovine α-herpesvirus 1
BRD: bovine respiratory disease
BRG1: Brahma-related gene 1
BRM: Brahma gene
ChIP: chromatin immunoprecipitation
co-IP: co-immunoprecipitation
CRM: *cis*-regulatory module
DEX: dexamethasone
GAPDH: Glyceraldehyde 3-phosphate dehydrogenase
GR: glucocorticoid receptor
GREs: glucocorticoid response elements
IEtu1: immediate early transcription unit 1
IP: immunoprecipitation
IR: internal repeat
LR: latency related
MEM: minimal essential media
MEM: minimal essential medium
RIPA: Radio-Immunoprecipitation Assay buffer
SWI/SNF: SWItch/Sucrose-NonFermentable
TBS-2: Tween 20
TG: trigeminal ganglia
TK: thymidine kinase
TR: terminal repeat
β-Gal: β-Galactosidase
